# Treatment Patterns and Survival among Adult Patients with Advanced Soft Tissue Sarcoma: A Retrospective Medical Record Review in the United Kingdom, Spain, Germany, and France

**DOI:** 10.1155/2018/5467057

**Published:** 2018-05-24

**Authors:** Saurabh P. Nagar, Daniel S. Mytelka, Sean D. Candrilli, Yulia D'yachkova, Maria Lorenzo, Bernd Kasper, Jose Antonio Lopez-Martin, James A. Kaye

**Affiliations:** ^1^RTI Health Solutions, 200 Park Offices Drive, Research Triangle Park, Durham, NC 27709, USA; ^2^Eli Lilly and Company, Lilly Corporate Center, Indianapolis, IN 46285, USA; ^3^Mannheim University Medical Center, Theodor-Kutzer-Ufer 1-3, 68167 Mannheim, Germany; ^4^12 de Octubre University Hospital, Avenida de Córdoba, s/n, 28041 Madrid, Spain

## Abstract

**Objective:**

To describe real-world treatment patterns and outcomes for patients with advanced soft tissue sarcoma (STS) not amenable to surgery or radiotherapy in the United Kingdom, Spain, Germany, and France.

**Methods:**

Physicians completed a web-based medical record abstraction for adult patients with advanced STS (other than Kaposi's sarcoma or gastrointestinal stromal tumor) who received ≥1 line of systemic therapy. Clinical characteristics, treatments, tumor responses, and mortality data were recorded.

**Results:**

A total of 130 physicians provided data for 807 patients. Patients' mean age at advanced STS diagnosis was 57.1 (±12.3) years; 59% were male. The most commonly identified histologic categories were leiomyosarcoma (28%), liposarcoma (13%), and rhabdomyosarcoma (11%). Overall, 57% of patients received only 1 line of therapy, 32% received 2 lines of therapy, and 11% received ≥3 lines of therapy. The most common first-line regimens were doxorubicin alone (41%), doxorubicin plus ifosfamide (19%), docetaxel plus gemcitabine (9%), paclitaxel alone (4%), and ifosfamide (4%). Median overall survival from start of treatment was estimated to be 17.6 months (95% confidence interval, 15.6–19.0 months).

**Conclusions:**

In real-world clinical practice, advanced STS is most commonly treated with older therapies in the United Kingdom, Spain, Germany, and France. New therapies that improve overall survival in advanced STS are needed.

## 1. Introduction

Soft tissue sarcoma (STS) refers to a rare and heterogeneous group of malignant tumors comprising more than 50 histologic subtypes that are derived from connective tissues and other cells of mesenchymal origin (e.g., fat, smooth or striated muscle, blood vessels, nerve sheath, subcutaneous tissue, and visceral connective tissue). Soft tissue sarcomas account for approximately 1% of all incident malignancies [1], with an estimated 23,574 new cases in Europe annually [2]. The estimated 5-year relative survival of patients with STS of any stage in Europe is 58%, and 5-year overall survival is approximately 50% [2, 3]. However, this rate is dependent on a number of factors, including the stage at diagnosis, histologic subtype, primary site, and presence of metastases [3].

An estimated 40% to 50% of patients with STS either present initially with metastatic or unresectable locally advanced disease (collectively, “advanced STS”) or present initially with a more limited extent of disease and subsequently develop advanced STS [4]. Although there are curative surgical options for early-stage STS, treatment goals for advanced STS are more limited. Chemotherapy (e.g., doxorubicin or ifosfamide, alone or in combination with each other or other agents) is most commonly used to treat patients with advanced STS [3, 5, 6]. The intent of these treatments generally is palliative rather than curative, and response rates are low (typically in the range of 10%–25%) [3].

The objective of this study was to evaluate treatment patterns and survival outcomes among patients with advanced STS not amenable to surgery or radiotherapy in real-world clinical settings in the United Kingdom (UK), Spain, Germany, and France in order to provide context for the evaluation of emerging therapies for this population.

## 2. Methods

### 2.1. Study Design

This study was a retrospective review of medical records of patients in the UK, Spain, Germany, and France treated with systemic therapy for advanced, histologically diagnosed STS (excluding Kaposi's sarcoma and gastrointestinal stromal tumor). Physicians abstracted anonymized data from medical records directly into a web-based data collection form. The relevant national competent authorities reviewed and approved the study on ethical grounds in all four countries. In Germany and Spain, the ethics committee at the study site of the principal investigator in each country reviewed and approved the study, as required by national authorities; this site-level approval applied to all participating sites within each country.

### 2.2. Study Population

#### 2.2.1. Physician Selection

Physicians specializing in oncology, who had been in practice for 3 to 35 years, and who personally treated at least 3 patients with advanced STS in the past year were recruited to provide patient data. Physician recruitment was done in partnership with country-specific agencies using in-house physician databases and physician directories maintained by local medical associations. Physicians were sampled from geographic regions throughout each country (5 regions in the UK, 5 in Germany, 4 in Spain, and 10 in France, with at least 1 physician representing each region). The dates of medical record abstraction in the UK were 7 August 2015 through 5 October 2015; in Germany, 20 August 2015 through 9 November 2015; in Spain, 21 August 2015 through 15 January 2016; and in France, 15 February 2016 through 4 May 2016.

#### 2.2.2. Patient Selection

Eligible patients were diagnosed with histologically confirmed metastatic or unresectable locally advanced STS (either at presentation or after progression from limited disease), between 1 January 2005 and 18 months before the initiation of record abstraction in the patient's country of residence. This end date for eligibility was chosen to allow the opportunity for a minimum duration of follow-up information unless a patient died sooner. Patients were required to be aged 18 years or older on the date of initial diagnosis of STS, to have started at least one line of systemic therapy for advanced STS, and to have records that were accessible to the abstracting physician for at least 1 year from diagnosis of advanced STS unless the patient died or refused further treatment.

Patients were excluded if they had received treatment with curative intent after diagnosis of advanced disease; received treatment with doxorubicin, daunorubicin, idarubicin, or other anthracyclines or anthracenediones (e.g., mitoxantrone) at any time before initiating first-line treatment for advanced STS; had evidence of concurrent malignancy, except adequately treated nonmelanoma skin cancer or in situ neoplasm; or had received first-line treatment within an interventional clinical trial (however, those who participated in post-first-line interventional trials or any noninterventional studies were eligible).

To reduce the possibility for physicians to select convenient patients, they were asked to select medical charts for patients whose last name begins with a randomly generated letter. If no patient whose last name began with that letter met the study criteria, physicians selected a patient whose last name began with the next letter in alphabetical order. To ensure internal consistency of the abstracted data, data checks for illogical or unusual patterns in physicians' responses on the data collection form were conducted.

### 2.3. Study Measures

Patient characteristics abstracted from the medical records included age, gender, race/ethnicity (except in France, where collection of such information is prohibited), comorbidities, and performance status. Tumor characteristics including American Joint Committee on Cancer cancer stage [7], histology, primary site, and metastatic sites were abstracted. For the analysis, specific histologic subtypes reported in the medical records (see Supplementary Appendix A) were grouped into broader categories based on the World Health Organization (WHO) Classification of Tumours of Soft Tissue and Bone [8].  Table 1 links the histologic categories used in the analyses to the specific histologic subtypes reported in medical records and to the broader histologic categories (chapter headings) in the WHO Classification of Tumours of Soft Tissue and Bone [8].

Data on STS treatments were collected from the time of diagnosis of advanced disease until the end of the most recent medical record data available. These included numbers and percentages of patients receiving cancer-directed treatments, detailed usage patterns for chemotherapy and targeted therapy (i.e., crizotinib, everolimus, imatinib, pazopanib, sirolimus, sorafenib, sunitinib, tamoxifen, and toremifene), and numbers and percentages of patients receiving supportive care during each line of systemic therapy and after stopping active treatment. Details of systemic therapy were collected, including regimens prescribed, dates and duration of therapy, and reasons for discontinuation. Performance status was recorded (if available) at the start of each treatment line. Tumor response and progression, according to Response Evaluation Criteria in Solid Tumors (RECIST) as interpreted by the physician, were also evaluated.

Mortality data, including death during follow-up and cause of death (i.e., STS related or not), were collected from medical records.

### 2.4. Statistical Analyses

All analyses were descriptive and exploratory in nature and conducted using SAS (version 9.4). No formal statistical tests comparing results across countries, histologic categories, treatment regimens, or lines of therapy were conducted. Results are presented as frequencies, proportions, means, and other summary measures of distributions as appropriate.

Overall survival was estimated using the Kaplan–Meier method, which accounts for right censoring for patients whose death was not recorded during the observation period; the date of censoring was the date of the last available medical encounter before the data abstraction.

## 3. Results

### 3.1. Physician Characteristics

A total of 130 physicians participated in the study (21 in the UK, 34 in Spain, 40 in Germany, and 35 in France). A majority (≥65%) of treating physicians practiced in a cancer center or academic/teaching hospital as their primary practice facility type. In the previous year, the participating physicians treated an average of 45 patients (standard deviation (SD), 37) with advanced STS.

### 3.2. Patient Characteristics

Overall, 807 patients were included in the study sample (199 in the UK, 203 in Spain, 204 in Germany, and 201 in France). The patients were 58.9% male (UK, 63.3%; Spain, 60.6%; Germany, 63.7%; France, 47.8%), and 92.7% of patients were white (UK, 89.5%; Spain, 99.0%; Germany, 89.7%; race/ethnicity not reported in France) (Table 2). Mean age at the time of advanced diagnosis was 57.1 years (SD, 12.3). Across countries, the most common histologic categories were leiomyosarcoma (28.4%), liposarcoma (13.0%), rhabdomyosarcoma (10.7%), vascular sarcoma (9.8%), fibroblastic/myofibroblastic sarcoma (9.7%), and synovial sarcoma (5.8%). In each country, leiomyosarcoma was the most commonly observed histologic category (UK, 30.7%; Spain, 22.2%; Germany, 32.4%; France, 28.4%).

The mean observed follow-up time (from diagnosis of advanced STS to death or the last medical encounter before data abstraction) was 24.7 months (SD, 20.9). Most patients were initially diagnosed with stage IV STS (*n*=557, 69.0% overall; 78.9% in the UK; 68.5% in Spain; 77.0% in Germany; 51.7% in France). Among patients initially diagnosed with limited-stage disease (*n*=188; 23.3% of the sample), the mean (SD) time from initial diagnosis of STS to development of advanced disease was 23.1 (20.6) months. Eight percent of patients were reported to have limited-stage disease at diagnosis but had the same dates of initial STS diagnosis and advanced STS diagnosis; these patients were considered to have advanced disease at diagnosis for the purposes of the analysis.

Overall, the lower extremity was the most common anatomical location of primary tumors (*n*=187; 23.2%). Among 229 patients with leiomyosarcoma, the most common primary site was the uterus (*n*=85; 37.1%). Histologic categories with relatively common primary tumors in other locations were liposarcoma in the retroperitoneum (33/105; 31.4%) and vascular sarcoma in the head or neck (16/79; 20.3%). Most patients had grade 3 histology (63.7%), primary tumor >5 cm (73.4%), and deep tissue invasion (81.2%). Among patients with distant metastasis, the most common sites of distant metastases were the lung (*n*=468; 70.8%) and liver (*n*=187; 28.3%).

The mean (SD) Eastern Cooperative Oncology Group performance status at the time of advanced STS diagnosis was 1.2 (0.6), and the mean Charlson Comorbidity Index score (excluding cancer items) was 1.0 (SD, 1.1) [9]. The most commonly occurring comorbidities were hypertension (*n*=259; 32.1%), depression (*n*=104; 12.9%), and diabetes without chronic complications or end-organ damage (*n*=102; 12.6%). The commonly occurring comorbidities were generally consistent across histologic categories. Among all patients, 346 (42.9%) had no comorbidities captured by the Charlson Comorbidity Index.

### 3.3. Treatment Patterns

#### 3.3.1. Overall Advanced STS Treatment

All patients received at least one line of systemic therapy for advanced STS as an eligibility criterion. The mean (SD) total number of lines of therapy received was 1.6 (0.8) (Table 3). Overall, 56.5% of patients received only one line of therapy, 32.5% received two lines of therapy, and 11.0% received at least three lines of therapy. Conventional chemotherapy alone was received by 84.7% of all patients, while targeted therapy alone was received by 2.5%; 12.8% received at least one drug in each class (data not shown).

Across all lines of therapy, the most commonly used drugs, as monotherapy or in combination, were doxorubicin (68.4%), ifosfamide (mesna was always coadministered with ifosfamide) (40.2%), gemcitabine (24.7%), and docetaxel (20.0%) (data not shown). The most commonly used drugs in the UK were doxorubicin (74.9%), ifosfamide (35.2%), gemcitabine (24.6%), and docetaxel (23.1%); in Spain, doxorubicin (59.6%), ifosfamide (37.0%), and gemcitabine (24.6%); in Germany, doxorubicin (74.5%), ifosfamide (39.2%), and gemcitabine (23.5%); and in France, doxorubicin (64.7%), ifosfamide (49.3%), gemcitabine (25.9%), and pazopanib (19.4%) (data not shown).

The most commonly used supportive/palliative medications included those used for pain control (57.7%), antiemetics (55.9%), and corticosteroids (25.7%); 21.3% of patients received growth factors (data not shown). Use of the cardioprotectant dexrazoxane was reported for only 3 patients (all in France).

#### 3.3.2. First-Line Systemic Therapy

Table 4 summarizes first-line treatment patterns overall and by country, and Table 5 summarizes first-line treatment patterns by histologic category. Overall, the most common first-line regimens were doxorubicin alone (40.9%), doxorubicin plus ifosfamide (19.0%), docetaxel plus gemcitabine (8.7%), paclitaxel alone (4.2%), and ifosfamide alone (4.1%). In the first-line setting, frequent use of paclitaxel for vascular sarcoma (35.4% (28 of 79)) and docetaxel plus gemcitabine (38.8% (33 of 85)) for uterine leiomyosarcoma was observed; doxorubicin monotherapy remained the most common regimen for nonuterine leiomyosarcoma (46.5% (67 of 144)). First-line treatments were generally similar across countries, with some notable exceptions. Trabectedin was used in the first-line setting in France, principally among patients with leiomyosarcoma (14.0% (8 of 57); data not shown); use in the other countries was more limited and generally did not occur until second line or later. Paclitaxel was often used for vascular sarcoma but less commonly in the UK (18.8% (3 of 16)) than in other countries (Spain 40.0% (10 of 25); Germany 64.7% (11 of 17); France 47.6% (10 of 21)) (data not shown). Across all the countries, the median number of cycles during the first-line treatment was 6 for aggregated chemotherapy and targeted therapy (data not shown).

The mean (SD) duration of first-line therapy ranged from 4.12 (2.14) months in Germany to 5.65 (3.19) months in Spain (see Supplementary Appendix B). Among histologic categories, the mean (SD) duration of first-line therapy was shortest in patients with liposarcoma (ranging from 3.53 (1.29) months in the UK to 5.49 (2.85) months in Spain) and longest in synovial sarcoma (ranging from 4.08 (2.28) months in Germany to 7.60 (2.18) months in France). Most patients discontinued first-line therapy because they had completed a planned course of treatment (55.8% overall, 73.4% in the UK, 37.0% in Spain, 66.7% in Germany, and 46.3% in France) or because of progressive disease (34.0% overall, 19.6% in the UK, 47.3% in Spain, 26.5% in Germany, and 42.3% in France) but very few (2.0% overall) for adverse events (Supplementary Appendix B).

#### 3.3.3. Second-Line Systemic Therapy

Table 6 presents the most common sequences of first-line and second-line regimens for the overall population. Most patients did not receive a second-line regimen, and treatment options were diverse for those who did. Monotherapy predominated, although patients who received first-line treatment with doxorubicin plus ifosfamide often received docetaxel plus gemcitabine as second-line therapy.

### 3.4. Outcomes

#### 3.4.1. Response Rates

Table 7 presents physician-reported objective response rates by first-line regimen. Response rates were greater for patients treated with doxorubicin plus ifosfamide and docetaxel plus gemcitabine (>50%) than for those treated with doxorubicin alone (37.9%) or ifosfamide alone (21.2%). Table 7 also presents response rates by histologic category. Objective response rates were greater for leiomyosarcoma and rhabdomyosarcoma (>50%) than for other histologic categories explored.

#### 3.4.2. Overall Survival

**Figure 1:**
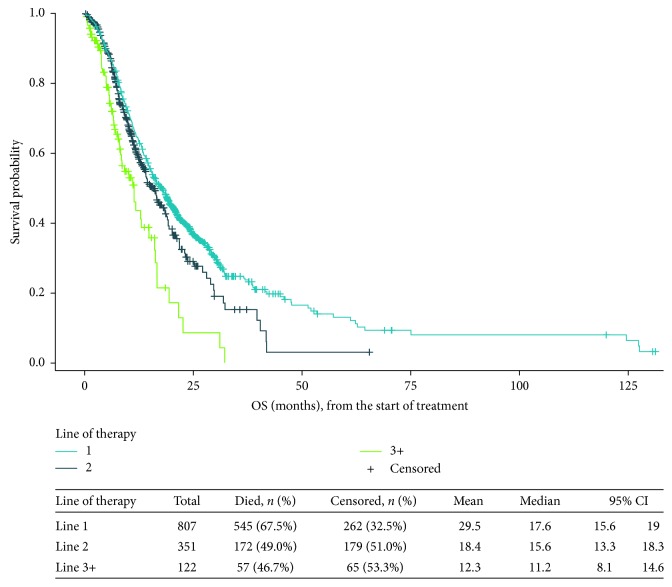
Overall survival (months) from initiation of each line of therapy. CI: confidence interval; OS: overall survival. *Note.* There is an overlap among the populations presented in the figure; for example, data for patients who initiated third-line therapy are included in each of these curves, but with different starting points. The survival distributions are not comparable because they include successively smaller subgroups of the study population and because time zero for each is the start of a specific line of therapy.

In total, 545 patients (67.5%) died during observed follow-up, and most (94.9%) of these deaths were STS related. The median estimated overall survival time from the start of first-line therapy was 17.6 months (95% confidence interval (CI), 15.6–19.0 months) (Figure 1). In successive subgroups of the study population receiving additional lines of treatment, the median overall survival was 15.6 months (95% CI, 13.3–18.3 months) from the start of second-line therapy and 11.2 months (95% CI, 8.1–14.6 months) from the start of third-line therapy (Figure 1).

**Figure 2:**
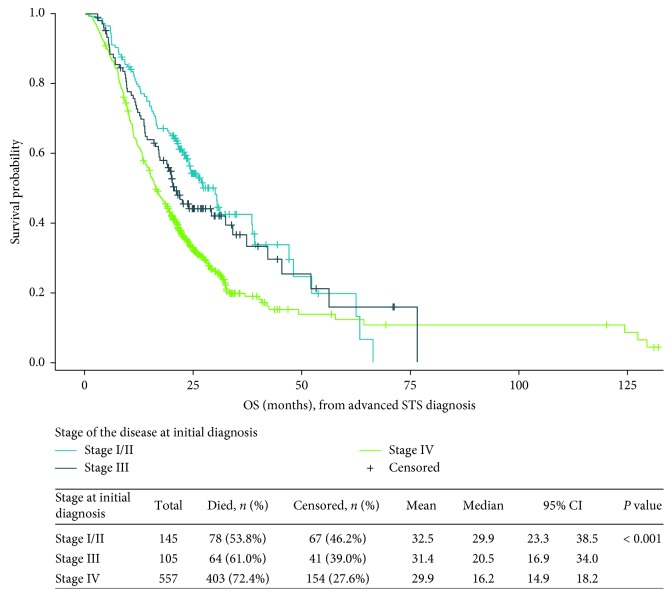
Overall survival (months) from the date of advanced STS diagnosis, by disease stage at initial diagnosis. CI: confidence interval; OS: overall survival. *Note.* Overall survival was estimated using the Kaplan–Meier method, which accounts for right censoring of the data for patients who were reported to have died during the observation period.

Median overall survival varied considerably by histologic category, ranging from 15.2 months (95% CI, 10.4–22.0 months) for patients with vascular sarcoma to 23.8 months (95% CI, 20.3–27.4 months) for those with leiomyosarcoma (Table 8). By disease stage at initial diagnosis, median overall survival from the time that advanced disease developed was 29.9 months (95% CI, 23.3–38.5 months) among patients with stage I or II disease at diagnosis, 20.5 months (95% CI, 16.9–34.0 months) among those with stage III disease, and 16.2 months (95% CI, 14.9–18.2 months) among those with stage IV disease at initial diagnosis (Figure 2).

## 4. Discussion

We have presented a descriptive review of treatment patterns among patients who received at least one line of systemic therapy for advanced STS in the UK, Spain, Germany, and France, based on a retrospective evaluation of their medical records. Owing to the observational nature of this study and the limited information available to control potential confounding, the results presented here are descriptive rather than suggestive of causal relationships between treatments and outcomes. Among the countries studied, there are differences in the organization of medical care and other cultural factors that could affect the treatment and survival of patients with advanced STS. The study provides recent data on characteristics, real-world treatment patterns, and survival from several European countries for a relatively large group of patients with advanced STS who received at least one line of systemic therapy.

The clinical characteristics of the study population are generally unsurprising. Consistent with other published information [2, 10], we found that leiomyosarcoma and liposarcoma were generally the most frequently reported histologic categories across study countries. As has been reported in literature [11, 12], our findings confirm that the lung is the most common location of metastases among patients undergoing systemic treatment for advanced STS. In our study, most primary tumors at the time of diagnosis of advanced STS were >5 cm and classified as “deep.” The relatively high proportion of patients with metastases at the time of initial diagnosis (relative to the population-based epidemiology of all patients diagnosed with STS) is likely due to the selection of patients for this study who ultimately had advanced disease and who underwent at least one line of systemic therapy.

Our study reported first-line treatment with doxorubicin alone or in combination as the most frequently used agent across the four countries we studied. Treatments were generally similar across histologic categories; however, some exceptions (in line with clinical guidelines) were noted, including the frequent first-line use of paclitaxel for vascular sarcoma and docetaxel plus gemcitabine for uterine leiomyosarcoma.

Treatment guidelines for the management of patients with STS in Europe have been published by a number of organizations, including the European Society for Medical Oncology (ESMO), the British Sarcoma Group, and the Spanish Group for Research on Sarcoma [13–15]. Results reported here are generally consistent with these guidelines in that standard first-line chemotherapy should be anthracycline based, with ifosfamide as a first-line alternative for patients who are not able to receive an anthracycline. Multiagent chemotherapy (gemcitabine plus docetaxel) has not been shown to provide superior results to single-agent doxorubicin [16]. Despite greater toxicity with doxorubicin plus ifosfamide than with doxorubicin alone, a higher response rate and longer progression-free survival with doxorubicin plus ifosfamide makes this combination a treatment of choice when tumor response is a high priority in patients with good performance status [17].

Ifosfamide (both as monotherapy and in combination with doxorubicin and/or dacarbazine) was among the five most commonly prescribed first-line treatments across all countries. Patients who received doxorubicin plus ifosfamide in the first line were also more likely to receive a second-line treatment than patients who received other first-line regimens.

Other real-world studies reported similar treatment patterns to what we present here. For example, Leahy et al. [18] conducted a retrospective medical record review of patients with metastatic STS across nine countries, including France, Germany, Spain, and the UK and found that the most common first-line regimens were doxorubicin monotherapy and anthracycline plus ifosfamide. Similarly, Nersesyan et al. [19] analyzed data for the European Union Five (France, Germany, Italy, Spain, and the UK) from country-specific cancer registries, published scientific studies, and proprietary surveys of 76 physicians conducted in March 2015. They concluded that doxorubicin plus ifosfamide is the most commonly used treatment in first line across the study countries, while trabectedin and pazopanib are used frequently in second- and third-line treatment (except in France, where first-line use of trabectedin among patients with leiomyosarcoma was not uncommon). Guest et al. [20] posit that “many clinicians do not initiate chemotherapy with ifosfamide monotherapy” at least in part because ifosfamide is associated with increased risks of toxicities at high doses (with high doses recommended after failure of first-line chemotherapy). Our data present somewhat conflicting findings in this regard, with ifosfamide monotherapy being one of the five most frequently used first-line therapies in both Spain and the UK (although not in France or Germany).

As Schöffski et al. [3] pointed out, “The heterogeneity of this disease poses a challenge to the physician caring for patients with sarcoma, as the prognosis and potential response to treatment in a given subtype of a mesenchymal malignancy is difficult to predict.” Evaluating the efficacy of treatments can be challenging given different histologic types, each of which may exhibit differential chemosensitivity [14] and response to treatments [21]. For example, in a recent study of patients in Japan with STS who were treated with pazopanib, Nakamura et al. [22] found significant differences in median progression-free survival across histologic subtypes, ranging from 8 weeks for liposarcoma to 18.6 weeks for leiomyosarcoma.

Previous studies have reported limited survival for patients with advanced STS [4, 23, 24], which varies somewhat by histologic category [25]. Our study confirms these findings, with median overall survival estimates of less than 2 years across histologic categories. It should be noted that in medical record reviews such as this, where the ability to control potential confounding is limited, it cannot be inferred that any observed survival differences among subgroups treated with different therapies are a result of treatment as opposed to differences in prognostic factors among patients who are prescribed particular therapies (“channeling bias”). It is apparent from our data, as well as from registry data and previously published studies, that survival rates for advanced STS are poor, indicating a continuing, critical need for advances in therapeutic options for this condition. With the exception of eribulin, which has demonstrated improved overall survival as a third-line treatment for STS [26], no new treatment has shown an overall survival improvement over the past 20 years.

A number of prior studies have evaluated symptom burden and medication use among adult patients with STS. In evaluating the literature, Chan et al. [27] found that pain, dyspnea, nausea, and vomiting were the most frequent symptoms experienced. Kuo et al. [28] conducted a study of an STS population in a UK sarcoma unit and found that more than half of assessed patients reported pain. Finally, Gough et al. [29] conducted a medical record abstraction in the UK and deduced that patients with metastatic STS have high symptom burden with pain being a significant problem. Our results are consistent with the literature in that more than half of all patients in our study (regardless of the country) utilized some form of pain medication.

Our results are subject to several limitations inherent to many retrospective medical record review studies. First, the patients selected for study inclusion represented a convenience sample in that the records were obtained from physicians who were willing to participate in the study. The extent to which physician self-selection for participation in studies such as this could influence results is unknown. Both the sampling procedures and the patient eligibility criteria limit the generalizability of this study. In particular, some types of STS are more common in younger patients (who were intentionally excluded from our study), and patients who were surgically cured and never developed advanced disease were excluded from this study. Thus, the study sample may not be completely representative of all patients in each country studied, and our findings may not be fully generalizable to the overall population of adult patients with advanced STS who undergo systemic treatment in these countries. Data were entered directly by the treating physicians based on medical records available at the time of data entry, and therefore the data are potentially subject to data entry errors and other limitations of retrospective data capture. Response rates observed in this study should be interpreted with caution. Response was subject to physicians' interpretation and may not have routinely been assessed radiographically; RECIST criteria may not have been as rigorously applied in routine practice settings as in clinical trials. Further, trabectedin and pazopanib were not commercially available during the full study period, and their utilization may have been underestimated.

## 5. Conclusions

This is one of the first large-scale, real-world retrospective medical record reviews to evaluate current treatment patterns and survival in a multinational population of adult patients with advanced STS. Patients in the UK, France, Germany, and Spain were generally treated consistently with established treatment guidelines. Variations in treatment patterns across countries were evident, but most involved use of anthracyclines; doxorubicin, alone or in combination with ifosfamide, was the most common first-line therapy. Median overall survival across histologic categories was less than 2 years, indicating a continuing, critical need for advances in therapeutic options for this difficult-to-treat disease.

## Figures and Tables

**Table 1:** Histologic categories.

Histologic category used in analysis	Histologic subtypes reported in the medical record	Corresponding WHO histologic category and subtypes^a^
Leiomyosarcoma	Smooth-muscle tumor and uterine leiomyosarcoma	Smooth-muscle tumor (i.e., leiomyosarcoma and leiomyoma of deep soft tissue)

Fibroblastic/myofibroblastic sarcoma	Fibroblastic/myofibroblastic sarcoma	Fibroblastic/myofibroblastic sarcoma (i.e., fibrosarcoma, myxofibrosarcoma, low-grade fibromyxoid sarcoma, and sclerosing epithelioid fibrosarcoma)

Liposarcoma	Adipocytic tumor	Adipocytic tumor (i.e., dedifferentiated liposarcoma, myxoid liposarcoma, and pleomorphic liposarcoma)

Vascular sarcoma	Vascular tumor of soft tissue and angiosarcoma	Vascular tumor (epithelioid hemangioendothelioma and angiosarcoma of soft tissue)

Rhabdomyosarcoma	Skeletal-muscle tumor	Skeletal-muscle tumor (i.e., rhabdomyosarcoma, including embryonal, alveolar, and pleomorphic forms, and spindle cell/sclerosing rhabdomyosarcoma)

Synovial sarcoma	Synovial sarcoma	Tumor of uncertain differentiation: synovial sarcoma

Others or not otherwise specified (NOS)	Chondro-osseous tumor, fibrohistiocytic tumor, pericytic tumor, nerve sheath tumor, tumor of uncertain differentiation, and undifferentiated/unclassified sarcoma	Not applicable So-called fibrohistiocytic tumor Perivascular tumor Chondro-osseous tumor Nerve sheath tumor Tumor of uncertain differentiation (unless otherwise noted^b^) Undifferentiated/unclassified tumor

WHO: World Health Organization; ^a^according to the WHO classification of tumours of soft tissue and bone [8]; ^b^synovial sarcoma, classified under “Tumours of uncertain differentiation,” was included in a separate histologic category for the analyses.

**Table 2:** Patient characteristics, overall and by country.

Characteristic	Overall (*N*=807)		UK (*n*=199)		Spain (*n*=203)		Germany (*n*=204)		France (*n*=201)	
	*n*	%	*n*	%	*n*	%	*n*	%	*n*	%
*Sex*										
Male	475	58.9	126	63.3	123	60.6	130	63.7	96	47.8
Female	332	41.1	73	36.7	80	39.4	74	36.3	105	52.2

*Age at advanced diagnosis (years)*										
Mean (SD)	57.1	12.3	56.5	12.4	56.6	13.1	57.4	11.0	57.9	12.6
Range (minimum, maximum)	21	90	21	90	21	85	28	77	22	84

*Ethnicity* ^a^										
White/Caucasian	562	92.7	178	89.5	201	99.0	183	89.7	—	—
African/Black	14	2.3	10	5.0	0	0	4	2.0	—	—
Asian or Pacific Islander	14	2.3	6	3.0	0	0	8	3.9	—	—
Middle eastern	8	1.3	2	1.0	2	1.0	4	2.0	—	—
Indian subcontinent	8	1.3	3	1.5	0	0	5	2.5	—	—

*Histologic category*										
Leiomyosarcoma	229	28.4	61	30.7	45	22.2	66	32.4	57	28.4
Others/NOS	183	22.7	40	20.1	35	17.2	43	21.1	65	32.3
Fibroblastic/myofibroblastic sarcoma	78	9.7	24	12.1	18	8.9	20	9.8	16	8.0
Liposarcoma	105	13.0	14	7.0	44	21.7	22	10.8	25	12.4
Vascular sarcoma	79	9.8	16	8.0	25	12.3	17	8.3	21	10.4
Rhabdomyosarcoma	86	10.7	33	16.6	20	9.9	21	10.3	12	6.0
Synovial sarcoma	47	5.8	11	5.5	16	7.9	15	7.4	5	2.5

*Stage of the disease at initial diagnosis*										
1A	1	0.1	0	0	0	0	0	0	1	0.5
1B	20	2.5	6	3.0	5	2.5	6	2.9	3	1.5
IIA	37	4.6	7	3.5	9	4.4	3	1.5	18	9.0
IIB	87	10.8	12	6.0	30	14.8	13	6.4	32	15.9
III (with no regional lymph node metastasis)	44	5.5	6	3.0	10	4.9	9	4.4	19	9.5
III (with regional lymph node metastasis)	61	7.6	11	5.5	10	4.9	16	7.8	24	11.9
IV	557	69.0	157	78.9	139	68.5	157	77.0	104	51.7
Number of patients where initial diagnosis was not an advanced stage^b^	188	23.3	25	12.6	54	26.6	27	13.2	82	40.8

*CCI score at advanced diagnosis* ^c^										
Mean (SD)	1.0	1.1	1.0	1.1	1.0	1.0	1.0	1.1	0.8	1.2
Range (minimum, maximum)	0	6	0	5	0	4	0	6	0	6

*Most common CCI conditions at advanced diagnosis* ^c^										
Depression	104	12.9	23	11.6	33	16.3	29	14.2	19	9.5
Diabetes without complications	102	12.6	20	10.1	40	19.7	30	14.7	12	6.0
Hypertension	259	32.1	64	32.2	63	31.0	74	36.3	58	28.9

*Anatomic location of primary tumor*										
Axilla	12	1.5	5	2.5	2	1.0	3	1.5	2	1.0
Breast	20	2.5	3	1.5	7	3.5	4	2.0	6	3.0
Lower extremity	187	23.2	42	21.1	53	26.1	53	26.0	39	19.4
Upper extremity	97	12.0	27	13.6	28	13.8	28	13.7	14	7.0
Head or neck	48	6.0	15	7.5	12	5.9	14	6.9	7	3.5
Gastrointestinal	38	4.7	16	8.0	5	2.5	12	5.9	5	2.5
Genitourinary	13	1.6	4	2.0	3	1.5	2	1.0	4	2.0
Gynecologic (other than uterus)	9	1.1	1	0.5	2	1.0	3	1.5	3	1.5
Mediastinum, lung, or pleura	26	3.2	4	2.0	9	4.4	7	3.4	6	3.0
Pelvis (nonvisceral)	65	8.1	23	11.6	13	6.4	17	8.3	12	6.0
Retroperitoneal	124	15.4	24	12.1	33	16.3	27	13.2	40	19.9
Trunk	67	8.3	16	8.0	11	5.4	18	8.8	22	11.0
Uterus	85	10.5	13	6.5	22	10.8	16	7.8	34	16.9
Unknown	13	1.6	6	3.0	1	0.5	0	0	6	3.0

*Site of metastasis among patients with distant metastasis*										
Bone	105	15.9	26	14.4	30	18.1	28	15.6	21	15.6
Brain	16	2.4	3	1.7	8	4.8	2	1.1	3	2.2
Distant lymph nodes	161	24.4	56	31.1	36	21.7	51	28.3	18	13.3
Exoskeletal	28	4.2	10	5.6	6	3.6	9	5.0	3	2.2
Liver	187	28.3	58	32.2	42	25.3	56	31.1	31	23.0
Lung	468	70.8	112	62.2	125	75.3	125	69.4	106	78.5
Mediastinum	38	5.8	11	6.1	11	6.6	8	4.4	8	5.9
Pericardium	9	1.4	2	1.1	3	1.8	3	1.7	1	0.7
Peritoneum	98	14.8	32	17.8	15	9.0	30	16.7	21	15.6
Pleura	42	6.4	8	4.4	12	7.2	9	5.0	13	9.6
Retroperitoneum	18	2.7	5	2.8	5	3.0	5	2.8	3	2.2
Others	5	0.8	1	0.6	0	0	0	0	4	3.0
Unknown	2	0.3	1	0.6	1	0.6	0	0	0	0

*ECOG PS at advanced diagnosis*										
PS 0	84	10.4	15	7.5	23	11.3	18	8.8	28	13.9
PS 1	474	58.7	162	81.4	86	42.4	144	70.6	82	40.8
PS ≥ 2	249	30.9	22	11.1	94	46.3	42	20.6	91	45.3

CCI: Charlson Comorbidity Index; ECOG: Eastern Cooperative Oncology Group; NOS: not otherwise specified; PS: performance status; SD: standard deviation; STS: soft tissue sarcoma; UK: United Kingdom; ^a^not collected in France; ^b^patients with stage I, II, or III at initial diagnosis and with time from initial to advanced diagnosis greater than zero; ^c^because the objective of the CCI score was to evaluate underlying comorbidity burden independent of STS, cancer was excluded from the CCI calculation for this study.

**Table 3:** General treatment utilization, overall and by country.

	Overall (*N*=807)		UK (*n*=199)		Spain (*n*=203)		Germany (*n*=204)		France (*n*=201)	
	*n*	%	*n*	%	*n*	%	*n*	%	*n*	%
*Total number of lines of therapy received*										
Mean (SD)	1.6	0.8	1.4	0.6	1.6	0.8	1.5	0.6	1.9	1.0

*Distribution*										
1	456	56.5	124	62.3	117	57.6	122	59.8	93	46.3
2	262	32.5	64	32.1	66	32.5	70	34.3	62	30.9
3+	89	11.0	11	5.5	20	9.9	12	5.9	46	22.9

SD: standard deviation; UK: United Kingdom.

**Table 4:** Five most commonly prescribed first-line treatment regimens, overall and by country.

Treatment	Overall (*N*=807)		UK (*n*=199)		Spain (*n*=203)		Germany (*n*=204)		France (*n*=201)	
	*n*	%	*n*	%	*n*	%	*n*	%	*n*	%
Doxorubicin	330	40.9	94	47.2	72	35.5	107	52.5	57	28.4
Doxorubicin + ifosfamide^a^	153	19.0	31	15.6	31	15.3	34	16.7	57	28.4
Docetaxel + gemcitabine	70	8.7	16	8.0	21	10.3	27	13.2	^b^	^b^
Paclitaxel	34	4.2	^b^	^b^	13	6.4	8	3.9	10	5.0
Ifosfamide^a^	33	4.1	15	7.5	10	4.9	^b^	^b^	^b^	^b^
Dacarbazine + doxorubicin + ifosfamide	^b^	^b^	11	5.5	^b^	^b^	^b^	^b^	^b^	^b^
Dacarbazine + ifosfamide^a^	^b^	^b^	^b^	^b^	^b^	^b^	6	2.9	^b^	^b^
Epirubicin + ifosfamide^a^	^b^	^b^	^b^	^b^	^b^	^b^	^b^	^b^	17	8.5
Trabectedin	^b^	^b^	^b^	^b^	^b^	^b^	^b^	^b^	11	5.5

NA: not applicable; UK: United Kingdom; ^a^mesna was always coadministered with ifosfamide; ^b^may have been observed in the overall or country-specific sample but was not among the five most commonly prescribed first-line regimens.

**Table 5:** Five most commonly prescribed first-line treatment regimens by histologic category.

Histologic category and treatment	*N* (%)
*Nonuterine leiomyosarcoma (n*=144)	
Doxorubicin	67 (46.5)
Doxorubicin + ifosfamide^a^	24 (16.7)
Docetaxel + gemcitabine	15 (10.4)
Ifosfamide^a^	9 (6.3)
Dacarbazine + doxorubicin + ifosfamide^a^	5 (3.5)

*Uterine leiomyosarcoma (n*=85)	
Docetaxel + gemcitabine	33 (38.8)
Doxorubicin + ifosfamide^a^	15 (17.6)
Doxorubicin	14 (16.5)
Trabectedin	7 (8.2)
Carboplatin + paclitaxel	2 (2.4)

*Fibroblastic/myofibroblastic sarcoma (n*=78)	
Doxorubicin	32 (41.0)
Doxorubicin + ifosfamide^a^	13 (16.7)
Ifosfamide^a^	6 (7.7)
Epirubicin + ifosfamide^a^	6 (7.7)
Dacarbazine + ifosfamide^a^	4 (5.1)

*Liposarcoma (n*=105)	
Doxorubicin	65 (61.9)
Doxorubicin + ifosfamide^a^	15 (14.3)
Docetaxel + gemcitabine	3 (2.9)
Docetaxel	3 (2.9)
Trabectedin	3 (2.9)

*Vascular sarcoma (n*=79)	
Paclitaxel	28 (35.4)
Doxorubicin	18 (22.8)
Doxorubicin + ifosfamide^a^	7 (8.9)
Ifosfamide^a^	4 (5.1)
Docetaxel + gemcitabine	3 (3.8)

*Rhabdomyosarcoma (n*=86)	
Doxorubicin	34 (39.5)
Doxorubicin + ifosfamide^a^	19 (22.1)
Ifosfamide^a^	5 (5.8)
Pazopanib	5 (5.8)
Docetaxel + gemcitabine	3 (3.5)

*Synovial sarcoma (n*=47)	
Doxorubicin	23 (48.9)
Doxorubicin + ifosfamide^a^	7 (14.9)
Ifosfamide^a^	4 (8.5)
Docetaxel + gemcitabine	3 (6.4)
Paclitaxel	3 (6.4)

*Others/NOS (n*=183)	
Doxorubicin	77 (42.1)
Doxorubicin + ifosfamide^a^	53 (29.0)
Docetaxel + gemcitabine	9 (4.9)
Epirubicin + ifosfamide^a^	7 (3.8)
Dacarbazine + doxorubicin + ifosfamide^a^	4 (2.2)

NOS: not otherwise specified; Denominators for the percentages are patients who received one of the five most commonly prescribed first-line regimens within each histologic category; ^a^mesna was always coadministered with ifosfamide.

**Table 6:** Treatment sequencing: most common first- and second-line treatment regimens.

First line	*N* (%)	Second line	*N* (%)
Doxorubicin	330 (40.9)	Ifosfamide^a^	63 (19.1)
		Pazopanib	24 (7.3)
		Trabectedin	19 (5.8)
		Docetaxel + gemcitabine	15 (4.5)
		Paclitaxel	5 (1.5)
		Others	16 (4.8)
		No treatment	188 (57)

Doxorubicin + ifosfamide^a^	153 (19.0)	Docetaxel + gemcitabine	38 (24.8)
		Pazopanib	21 (13.7)
		Trabectedin	17 (11.1)
		Others	23 (15.0)
		No treatment	54 (35.3)

Docetaxel + gemcitabine	70 (8.7)	Doxorubicin	13 (18.6)
		Pazopanib	7 (10)
		Doxorubicin + ifosfamide^a^	4 (5.7)
		Ifosfamide^a^	3 (4.3)
		Others	9 (12.9)
		No treatment	34 (48.6)

Paclitaxel	34 (4.2)	Doxorubicin	3 (8.8)
		Ifosfamide^a^	1 (2.9)
		No treatment	30 (88.2)

Ifosfamide-mesna	33 (4.1)	Doxorubicin	3 (9.1)
		Others	3 (9.1)
		No treatment	27 (81.8)

^a^Mesna was always coadministered with ifosfamide.

**Table 7:** Response rates by first-line regimen and histologic category, overall.

Characteristic	Best response according to RECIST^a^			
	Complete response	Partial response	Stable disease	No response/disease progression
*First-line regimen, n (%)*				
Doxorubicin (*n*=330)	5 (1.5)	120 (36.4)	115 (34.9)	87 (26.4)
Doxorubicin + ifosfamide^b^ (*n*=153)	14 (9.2)	71 (46.4)	40 (26.1)	28 (18.3)
Docetaxel + gemcitabine (*n*=70)	3 (4.3)	35 (50.0)	18 (25.7)	14 (20.0)
Paclitaxel (*n*=34)	5 (14.7)	9 (26.5)	6 (17.7)	13 (38.2)
Ifosfamide^b^ (*n*=33)	0	7 (21.2)	14 (42.4)	11 (33.3)
All other regimens (*n*=186)	18 (9.6)	71 (38.0)	54 (28.9)	43 (23.0)

*Histologic category* ^*c*^ *n (%)*				
Leiomyosarcoma (*n*=229)	15 (6.6)	104 (45.4)	66 (28.8)	44 (19.2)
Others/NOS (*n*=183)	7 (3.8)	70 (38.3)	52 (28.4)	52 (28.4)
Fibroblastic/myofibroblastic sarcoma (*n*=78)	5 (6.4)	24 (30.8)	34 (43.6)	15 (19.2)
Liposarcoma (*n*=105)	4 (3.8)	34 (32.4)	32 (30.5)	32 (30.5)
Vascular sarcoma (*n*=79)	8 (10.1)	25 (31.7)	22 (27.9)	23 (29.1)
Rhabdomyosarcoma (*n*=86)	5 (5.8)	41 (47.7)	22 (25.6)	18 (20.9)
Synovial sarcoma (*n*=47)	1 (2.1)	15 (31.9)	19 (40.4)	12 (25.5)

NOS: not otherwise specified; RECIST: Response Evaluation Criteria in Solid Tumors; ^a^among patients with known response; ^b^mesna was always coadministered with ifosfamide; ^c^response rates are for first-line regimens administered in each histologic category.

**Table 8:** Overall survival (months) by histologic category.

Histologic category	Total	Died, *n* (%)	Censored, *n* (%)	Mean	Median	95% CI	
Leiomyosarcoma	229	132 (57.6)	97 (42.4)	27.2	23.8	20.3	27.4
Others/NOS	183	134 (73.2)	49 (26.8)	20.5	16.2	14.2	18.4
Fibroblastic/myofibroblastic sarcoma	78	55 (70.5)	23 (29.5)	19.7	19.2	13.5	22.8
Liposarcoma	105	78 (74.3)	27 (25.7)	34.8	16.3	13.3	21.0
Vascular sarcoma	79	54 (68.4)	25 (31.6)	26.7	15.2	10.4	22.0
Rhabdomyosarcoma	86	57 (66.3)	29 (33.7)	27.4	20.1	13.1	22.3
Synovial sarcoma	47	35 (74.5)	12 (25.5)	23.5	16.2	12.6	23.2

CI: confidence interval; NOS: not otherwise specified.

## Supplementary Materials

Supplementary 1 Supplementary Appendix A: summarizes the histologic subtype categories for cellular classification of tumors at initial diagnosis of STS, as reported in medical records, that were used in the analyses.

Supplementary 2 Supplementary Appendix B: provides detailed information on patient characteristics and treatment patterns, overall, and by country.
