# Supplementary material for: Treatment Patterns and Survival among Adult Patients with Advanced Soft Tissue Sarcoma: A Retrospective Medical Record Review in the United Kingdom, Spain, Germany, and France
Source: Sarcoma. 2018 May 24;2018:5467057. doi: 10.1155/2018/5467057 (PMC5994280; doi:10.1155/2018/5467057)
Supplement: Supplementary 1 — Supplementary Appendix A: summarizes the histologic subtype categories for cellular classification of tumors at initial diagnosis of STS, as reported in medical records, that were used in the analyses. [file 5467057.f1.docx]

# SupplementaL Appendix A

## Cellular Classification of the Tumor at Initial Diagnosis of STS Reported in Medical Records

| Adipocytic tumor  *(i.e., dedifferentiated liposarcoma, myxoid liposarcoma, pleomorphic liposarcoma)* |
| --- |
| Angiosarcoma |
| Chondro-osseous tumor *(i.e., extraskeletal osteosarcoma)* |
| Fibroblastic/myofibroblastic tumor *(i.e., fibrosarcoma, myxofibrosarcoma, low-grade fibromyxoid sarcoma, sclerosing epithelioid fibrosarcoma)* |
| Fibrohistiocytic tumor *(i.e., plexiform fibrohistiocytic tumor, giant cell tumor of soft tissue, malignant tenosynovial giant cell tumor)* |
| Skeletal-muscle tumor *(i.e., rhabdomyosarcoma, including embryonal, alveolar, and pleomorphic forms, spindle cell/sclerosing rhabdomyosarcoma)* |
| Smooth-muscle tumor *(i.e., leiomyosarcoma, excluding uterine leiomyosarcoma)* |
| Pericytic (perivascular) tumor (*i.e., malignant glomus tumor*) |
| Vascular tumor of soft tissue  (*i.e., epithelioid hemangioendothelioma*) |
| Nerve sheath tumor *(i.e., perineurioma, malignant peripheral nerve sheath tumor, epithelioid malignant peripheral nerve sheath tumor, malignant triton tumor, malignant granular cell tumor, ectomesenchymoma)* |
| Synovial sarcoma |
| Tumor of uncertain differentiation *(i.e., epithelioid sarcoma, alveolar soft-part sarcoma, clear cell sarcoma of soft tissue, extraskeletal myxoid chondrosarcoma, extraskeletal Ewing sarcoma, desmoplastic small round cell tumor, extra-renal rhabdoid tumor, PEComa, intimal sarcoma)* |
| Uterine leiomyosarcoma |
| Undifferentiated/unclassified sarcoma *(i.e., undifferentiated spindle cell sarcoma, undifferentiated pleomorphic, undifferentiated round cell sarcoma, undifferentiated epithelioid sarcoma, undifferentiated sarcoma NOS)* |

NOS = not otherwise specified; PEComa = perivascular epithelioid cell tumor; STS = soft tissue sarcoma.
